# A Fixed-End Beam–Cantilever Piezoelectric MEMS Speaker with Flexible Supporting Layer

**DOI:** 10.3390/mi17020215

**Published:** 2026-02-05

**Authors:** Guanzong Shao, Yujiang Li, Zhiyong Hu, Qi Wang, Jinshi Zhao

**Affiliations:** 1 School of Integrated Circuit Science and Engineering, Engineering Research Center of Optoelectronic Devices and Communication Technology, Ministry of Education, State Key Laboratory of Crystal Materials, Tianjin University of Technology, Tianjin 300384, China; sgz0701@stud.tjut.edu.cn (G.S.); liyujiang0714@163.com (Y.L.); 2 National Key Laboratory of Advanced Micro and Nano Manufacture Technology, Shanghai Jiao Tong University, Shanghai 200240, China; huzhiyonghzy@sjtu.edu.cn

**Keywords:** piezoelectric, MEMS, speaker, fixed-end beam–cantilever, flexible supporting layer

## Abstract

Conventionally designed piezoelectric micro-electro-mechanical systems (MEMS) speakers with thin-film-type, piston-type, and cantilever-type vibration membranes still adopt a Si supporting layer, which not only hinders the improvement of sound pressure level (SPL) but also lacks characterization of reliability. In this paper, we propose a fixed-end beam–cantilever piezoelectric MEMS speaker with a flexible supporting layer, achieving an SPL comparable to that of traditional three types of piezoelectric MEMS speakers with a Si supporting layer, and displaying good reliability. The measured results performed on encapsulated prototypes mounted to an acoustic test adaptor demonstrate that under a driving voltage of 1 V_rms_, the SPL exceeds 51.6 dB in the human audible frequency range of 20 Hz–20 kHz, the total harmonic distortion (THD) remains below 3.4% above 430 Hz, satisfying the basic requirements for human auditory perception. Moreover, further experiments also prove its reliability by revealing no abnormal sound output, no fracture after being dropped from heights of 1 to 5 m, and the retention of over 92% SPL following 100 h of continuous music playback. This fixed-end beam–cantilever piezoelectric MEMS speakers with a flexible supporting layer provide researchers and enterprises with brand-new design ideas and a fresh perspective, which may potentially promote their development and practical application.

## 1. Introduction

Speakers are electroacoustic transducers that convert electrical signals into acoustic signals, widely present in various aspects of life, such as mobile phones, headphones, computers, human–computer interaction, and the Internet of Things (IoT) [1,2,3]. To meet the basic auditory needs of the human ear, speakers must cover the frequency range from 20 Hz to 20 kHz. Traditional speakers easily satisfy the basic hearing requirements of humans; challenges include large size, high cost, complex manufacturing processes, and difficult integration [4,5,6]. Likewise, wearable or portable audio devices, constrained by physical size, must use miniature speakers to reproduce the full audio range of the human ear, introducing additional limitations and affecting sound quality across the entire frequency range. The rapid advancement of micro-electromechanical systems (MEMS) technology has broken the boundary between traditional mechanics and electronics, not only achieving miniaturization and electronization of mechanical components but also improving product performance and reliability while reducing production costs and energy consumption [7]. This technological innovation gives MEMS speakers the potential to end the market dominance of traditional devices [8,9,10,11,12].

The electromagnetic MEMS speakers are realized based on Faraday’s law of electromagnetic induction, which utilizes the magnetic field generated by an electric current passing through a coil, causing the magnetic poles of the formed electromagnet to interact with those of a permanent magnet. This interaction pushes the membranes to vibrate, converting electrical current signals into sound signals. However, the manufacturing and assembly processes of the metal coil are relatively complex, and the magnet block is large, making integration and miniaturization difficult [13,14,15]. Electrostatic MEMS speakers use electrostatic forces to enable two independent diaphragms to vibrate and radiate sound waves. Although they offer advantages such as an extremely light diaphragm mass and excellent music reproduction capability, they face limitations of the pull-in effect and high driving voltage requirements [16,17,18]. Piezoelectric MEMS speakers operate based on the inverse piezoelectric effect, in which mechanical vibration is induced to push air and propagate sound waves. In comparison, piezoelectric MEMS speakers featuring miniaturization, theoretically high sound pressure level (SPL), and easy integration can address the aforementioned issues encountered by conventional ones [19,20,21,22,23]; this is expected to spearhead a revolutionary transformation in the audio field [12,20,24,25,26,27,28,29].

However, the reported piezoelectric MEMS speakers still fail to meet the SPL requirement of commercial viability and lack reliability testing. To solve these problems, Wang et al. [30] fabricated a circular film-type piezoelectric MEMS speaker with a Si supporting layer, which achieved about 60 dB SPL in the frequency range of 20 Hz–20 kHz under a driving voltage of 10 V_pp_. Cheng et al. [31] designed a piezoelectric MEMS speaker with a spiral spring design, enhancing the SPL in low-frequency ranges by increasing the length of the spiral spring while achieving improved out-of-plane vibration displacement and SPL through dual driving electrodes. The speaker achieves 50 dB SPL in the frequency range of 20 Hz–20 kHz under a driving voltage of 2 V_pp_. We developed a cantilever-type piezoelectric MEMS speaker in our previous work by implementing a rigid–flexible coupling membrane to eliminate acoustic losses while preserving the large vibration displacement characteristic of cantilever structures. Under a driving voltage of 2 V_rms_, this speaker achieved about 59 dB SPL in the frequency range of 20 Hz–20 kHz [32]. Furthermore, the devices reported in these studies lack reliability testing. Consequently, the inadequate SPL and the absent reliability tests exhibited by piezoelectric MEMS speakers have hindered the process of commercialization.

Here, we present a fixed-end beam–cantilever piezoelectric MEMS speaker based on a flexible supporting layer, where rectangular cantilevers are symmetrically distributed along both sides of a fixed-end beam. Test results obtained in an acoustic test adapter demonstrate that when the driving voltage is 1 V_rms_, an SPL higher than 51.6 dB is achieved in the frequency range of 20 Hz–20 kHz, which is comparable to the SPL of the three conventional types of piezoelectric MEMS speakers with Si supporting layers. Additionally, the total harmonic distortion (THD) is less than 3.4% and higher than 430 Hz, meeting the auditory requirements of the human ear. Furthermore, the extended experiments verified its reliability by demonstrating no abnormal sound, no fracture after being dropped from a height of 1–5 m, and an SPL retention rate of over 92% after playing music for 100 h continuously. The designed fixed-end beam–cantilever structure can enhance the SPL, and the adoption of a mica sheet can effectively improve reliability, both of which significantly contribute to advancing the practical development of piezoelectric MEMS speakers.

## 2. Design, Simulation, and Preparation Process

### 2.1. Design Strategy

**Figure 1:**
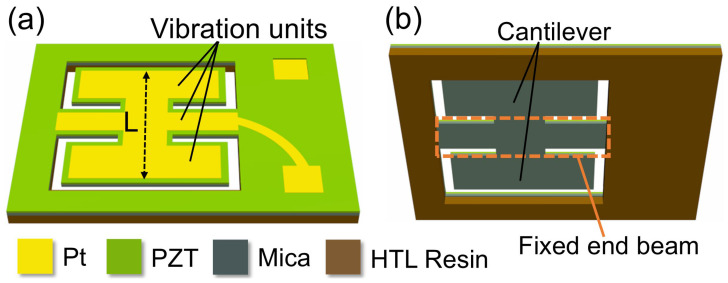
Structure and design of the proposed fixed-end beam–cantilever piezoelectric MEMS speaker with a flexible supporting layer: (**a**) overall structure; (**b**) back cavity.

Some butterflies can flap their wings with large amplitude, and their torsos exhibit an upward bending change when flapping upward [33,34,35,36,37]. Thus, the deformation degrees of the wings and torso can be superimposed. Inspired by this characteristic, the inverse piezoelectric effect is utilized to imitate the control of butterfly wings and torso. The two symmetric cantilevers and one fixed-end beam are designed to imitate the butterfly wings and torso, respectively, translating biomechanical dynamics into structural dynamics. Therefore, the superposition of the deformations of the cantilevers and fixed-end beam is employed to enhance the vibration amplitude, leading to an improvement in the SPL. Therefore, we demonstrate a fixed-end beam–cantilever piezoelectric MEMS speaker based on a flexible supporting layer [38,39], where two cantilevers are symmetrically bound to both sides of a central fixed-end beam, and the entire vibration units are distributed within a square with a side length (*L*) of 4 mm, as shown in Figure 1a. The back cavity is shown in Figure 1b; the two rectangular cantilever beams are attached to both sides of the fixed-end beam, with the two ends of the fixed-end beam anchored to the support frame. Compared with flexible materials such as PI (with a Young’s modulus of about 2–5 GPa), the mica sheet (with a Young’s modulus of about 60 GPa) has higher rigidity, which can ensure the flatness of the membrane. Compared with the Si supporting layer (with a Young’s modulus of about 130 GPa), the mica sheet has lower rigidity while still being able to guarantee the flatness of the membrane. Therefore, the adoption of the flexible supporting layer can increase the vibration displacement and SPL under low voltage. Meanwhile, the mica can also maintain good fracture resistance and fatigue resistance of the device, mainly due to its low elastic modulus, layered structure, and certain flexibility. Furthermore, in contrast to traditional Si supporting-layer materials that require deposition of a SiO_2_ insulation layer, a mica sheet can simultaneously function as both a supporting layer and an insulating layer.

### 2.2. Simulations

**Figure 2:**
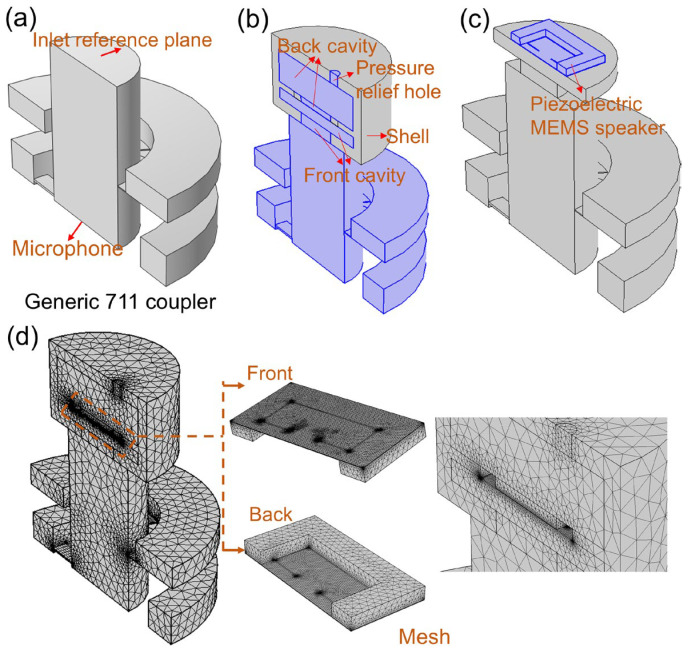
The generic 711 coupler and the simulation model. (**a**) The generic 711 coupler. (**b**) The simplified outer shell. (**c**) The fixed position of the proposed fixed-end beam–cantilever piezoelectric MEMS speaker. (**d**) The mesh setting of the proposed fixed-end beam–cantilever piezoelectric MEMS speaker and the generic 711 coupler.

COMSOL 6.4 Multiphysics software was used for simulations. The material properties are shown in Table 1. The generic 711 coupler and simulation model are shown in Figure 2. Figure 2a demonstrates the generic 711 coupler, which can be used to simulate acoustic characteristics of a standard human ear canal. The generic 711 coupler approximates the acoustic transfer impedance inside the ear canal from the tip of the earplug (ear insert or ear mold) at the reference plane to the eardrum. Therefore, the device should have the same acoustic characteristics as the average occluded human ear canal and eardrum system, roughly from the second bend of the ear canal to the eardrum, and is commonly used to measure hearing aids and headphones that are coupled to the human ear via insert earphones. The coupler measurements do not include sound leakage between the ear mold and the ear canal. At the same time, because the coupler represents a normal, average human ear, it does not simulate the large variations in acoustic performance that exist among different individual ears. As shown in Figure 2b, it displays a simplified outer shell in the simulation model, including the front cavity, back cavity, and pressure-relief hole. The fixed position of the proposed piezoelectric MEMS speaker in the simulation model is shown in Figure 2c, with the upper surface of its vibration membrane facing the inlet reference plane [40,41]. The mesh setting of the proposed fixed-end beam–cantilever piezoelectric MEMS speaker is shown in Figure 2d, which adopts a free tetrahedron structure. The mesh setting of the generic 711 coupler is also shown in Figure 2d.

**Figure 3:**
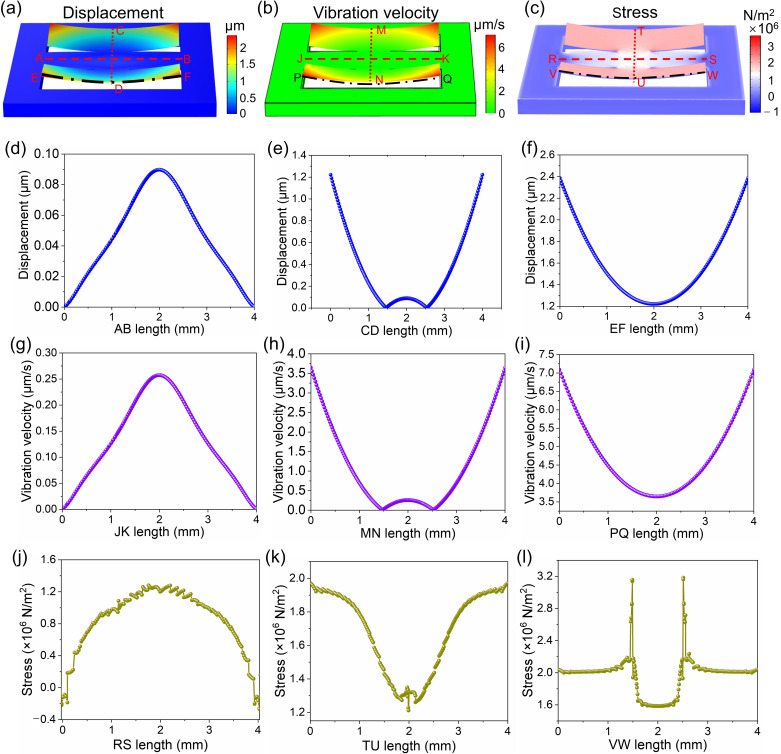
Simulations of the vibration displacement and vibration velocity distributions of the vibration membrane at 20 Hz under a driving voltage of 1 V_rms_. (**a**) The vibration displacement distribution simulation of the vibration membrane. (**b**) The vibration velocity distribution simulation of the vibration membrane. (**c**) The stress distribution simulation of the vibrating membrane. The displacement distribution simulation of the AB (**d**), CD (**e**), and EF (**f**) lines on the upper surface of the membrane cross-section. The vibration velocity distribution simulation of the JK (**g**), MN (**h**), and PQ (**i**) lines on the upper surface of the membrane cross-section. The stress distribution simulation of the RS (**j**), TU (**k**), and VW (**l**) lines on the upper surface of the membrane cross-section.

The boundary conditions at both ends of the fixed-end beam are constrained, and the driving voltage is 1 V_rms_. The vibration displacement distribution simulation of the vibration membrane at 20 Hz is shown in Figure 3a, with the regions of higher vibration displacement distributed along the non-constrained edges of the two cantilevers. The vibration velocity distribution simulation of the vibration membrane at 20 Hz under a driving voltage of 1 V_rms_ is shown in Figure 3b. The higher vibration velocity regions are concentrated at the edges of the two cantilevers. The stress distribution simulation of the vibration membrane at 20 Hz under a driving voltage of 1 V_rms_ is shown in Figure 3c. The stress undergoes a sign change at the edge of the fixed-end beam. The displacement distribution of the AB, CD, and EF lines on the upper surface of the membrane cross-section is shown in Figure 3d–f. The maximum displacement on the fixed-end beam reaches about 90 nm, while it reaches about 2.4 μm on the cantilever. The vibration velocity distribution of the JK, MN, and PQ lines on the upper surface of the membrane cross-section is shown in Figure 3g–i. The maximum vibration velocity regions are also located at the edges of the two cantilevers, reaching approximately 7 μm/s. The stress distribution of the RS, TU, and VW lines on the upper surface of the membrane cross-section is shown in Figure 3j–l. As shown in Figure 3j, a sign change in stress occurs within an approximately 130 μm-wide region at the edge of the fixed-end beam. Therefore, this region was taken into account when designing the electrode layout. In addition, because ablation occurs at the edges during the laser engraving process, the distance between the top electrode and the edge was set to 200 μm.

**Figure 4:**
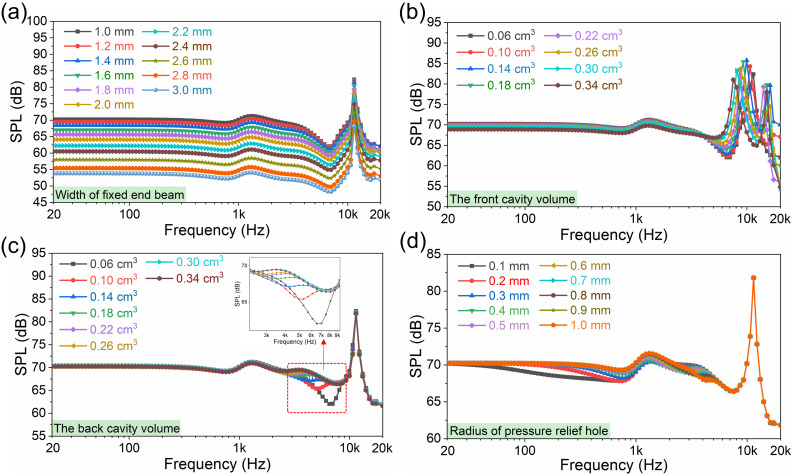
SPL simulations of the proposed fixed-end beam–cantilever piezoelectric MEMS speaker based on a flexible supporting layer at 1 V_rms_ under 20 Hz–20 kHz: (**a**) the effect of a fixed-end beam with different widths on SPL curves in simulation; (**b**) the effect of different front cavity volumes on SPL curves in simulation; (**c**) the effect of different back cavity volumes on SPL curves in simulation; and (**d**) the effect of a pressure-relief hole with different radii on SPL curves in simulation.

The SPL simulations of the proposed fixed-end beam–cantilever piezoelectric MEMS speaker with flexible supporting layer at 1 V_rms_ under 20 Hz–20 kHz are displayed in Figure 4. The simulation in Figure 4a shows the effect of a fixed-end beam with different widths on SPL curves. It can be seen that as the width of the fixed-end beam increases from 1 mm to 3 mm, the SPL decreases. Considering that the narrow beam may break during vibration, 1 mm was chosen as the width of the fixed-end beam. The volumes of the front and back cavities have a significant effect on the SPL curve. Figure 4b shows the SPL curves of the proposed piezoelectric MEMS speaker with different front cavity volumes. As the front cavity volume increases from 0.06 cm^3^ to 0.34 cm^3^, the resonance frequency exhibited by SPL curves shows a decreasing trend, and the SPL gradually becomes smaller within about 20 Hz–3 kHz. In the high-frequency range of 16–20 kHz, SPL shows a trend of first decreasing and then increasing as the front cavity volume increases from 0.06 cm^3^ to 0.34 cm^3^; the increase in front cavity volume primarily causes the first-order vibration frequency to shift forward, leading to the second-order vibration frequency appearing within the full-frequency range of 20 Hz to 20 kHz. Therefore, 0.06 cm^3^ was chosen as the front cavity volume of the device. Figure 4c shows SPL curves of the proposed piezoelectric MEMS speaker with different back cavity volumes. With the back cavity volume increasing from 0.06 cm^3^ to 0.34 cm^3^, the SPL only undergoes significant changes at approximately 3 kHz to 8 kHz. Additionally, at high frequencies near 20 kHz, when the back cavity volume increases, the SPL decreases significantly. Therefore, 0.26 cm^3^ was chosen as the back cavity volume of the device. The SPL curves simulation of the proposed piezoelectric MEMS speaker with pressure-relief holes of different radii is shown in Figure 4d. When the radius of the pressure-relief hole increases from 0.1 mm to 1 mm, there is no change in the SPL within the frequency range of 6–20 kHz. Significant changes are only observed in the frequency range of approximately 20 Hz–6 kHz, and there are alterations in the numerical magnitude relationship of the SPLs between 2 kHz and 5 kHz. Therefore, a radius of 0.5 mm was chosen for the pressure-relief hole.

**Figure 5:**
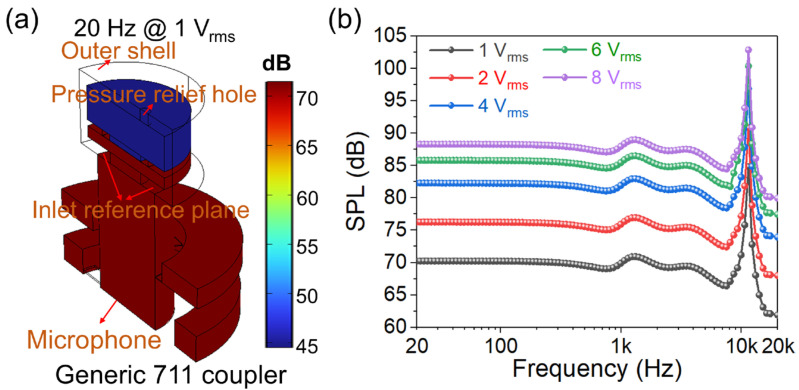
The SPL simulation of the proposed fixed-end beam–cantilever piezoelectric MEMS speaker in a generic 711 coupler: (**a**) the SPL distribution at 20 Hz in a generic 711 coupler under 1 V_rms_; (**b**) the SPL curves at different driving voltages.

Figure 5 demonstrates the SPL simulation of the proposed fixed-end beam–cantilever piezoelectric MEMS speaker in a generic 711 coupler. Figure 5a illustrates the SPL distribution of the proposed fixed-end beam–cantilever piezoelectric MEMS speaker in a generic 711 coupler at 20 Hz under a driving voltage of 1 V_rms_. It can be observed that the SPL achieves about 71 dB in the coupler. Figure 5b shows the SPL curves of the proposed fixed-end beam–cantilever piezoelectric MEMS speaker in the generic 711 coupler under different driving voltages. The simulation results indicate that under a driving voltage of 1 V_rms_, the SPL exceeds 61.8 dB within the frequency range of 20 Hz–20 kHz. As the driving voltage increases, the SPL rises, but the increment gradually decreases.

### 2.3. Preparation Process

**Figure 6:**
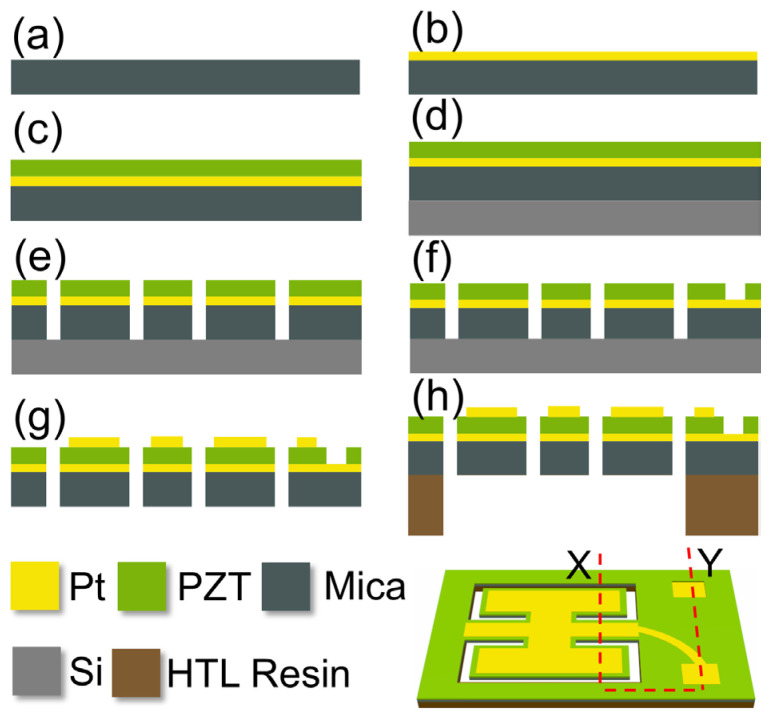
The process flow of the fixed-end beam–cantilever piezoelectric MEMS speaker based on a flexible supporting layer: (**a**) cleaning the mica sheet; (**b**) sputtering the lower electrode; (**c**) preparing the PZT thin film; (**d**) bonding the mica to the wafer surface; (**e**) engraving the overall center-bound pattern; (**f**) wet-etching the PZT; (**g**) sputtering the upper electrode; and (**h**) bonding the chip to the HTL resin.

The schematic diagram of the experimental steps is presented along the XY cross-section of the chip in Figure 6. A mica sheet with a thickness of about 25 μm was selected and cleaned thoroughly (Figure 6a). About 100 nm-thick Pt was deposited on the upper surface of the mica sheet by magnetron sputtering as the lower electrode (Figure 6b). Subsequently, the PZT (52/48) precursor solution, with a composition located on the morphotropic phase boundary, was supplied by Mitsubishi Materials Corporation. The precursor solution with a concentration of 0.5 mol/L was spin-coated onto the Pt surface at 1500 rpm (Figure 4c) [42,43,44,45,46,47], held at 300 °C for 20 min in an air atmosphere, and then annealed at ~650 °C in an air atmosphere for 1 h. Thus, a ~1 μm PZT thin film was prepared through a sol–gel process as the functional material layer. After the upper surface of a wafer with a thickness of approximately 430 μm was cleaned, photoresist was coated onto it. The mica sheet was then gently placed on the photoresist surface, followed by lightly pressing a glass sheet onto the mica sheet to ensure its flatness. Once the photoresist dried naturally, the mica sheet bonded to the wafer surface (Figure 6d). Then, the fixed-end beam–cantilever pattern was carved out by a laser engraving machine (Figure 6e). Next, the PZT was selectively etched according to the pre-designed pattern to expose the lower electrode (Figure 6f) [32], followed by the sputtering of approximately 100 nm-thick Pt layer on the PZT surface as the upper electrode. And then, it was immersed in N-Methylpyrrolidone (NMP) solution and subjected to ultrasonic treatment. The photoresist was dissolved, causing the separation of the wafer and the chip. The chip was then dried on a hot plate (Figure 6g). Then, the chip was softly clamped with silicone soft-tipped tweezers and placed on a 3D-printed HTL resin frame, which was fixed with ultraviolet (UV) curing adhesive and cured under UV light irradiation (Figure 6h). Finally, the chip was mounted on a printed circuit board (PCB), wires were connected between the chip and PCB, and the PCB was fixed inside a 3D-printed enclosure.

## 3. Results and Discussion

### 3.1. Photos and SEM Images

**Figure 7:**
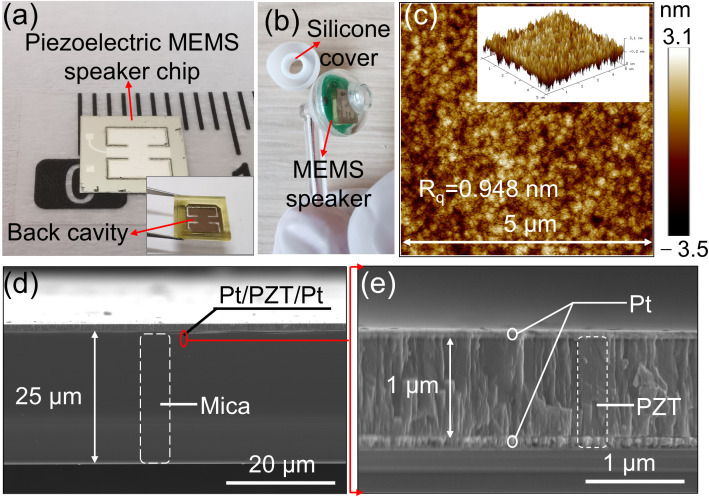
Fabricated fixed-end beam–cantilever piezoelectric MEMS speaker chip, PZT surface roughness, and SEM images: (**a**) MEMS speaker chip; (**b**) packaged device; (**c**) PZT surface roughness; (**d**) cross-section view of the vibration membrane; and (**e**) cross-section view of the PZT film.

The fabricated piezoelectric MEMS speaker chip is shown in Figure 7a. Two rectangular cantilevers are bound to both sides of a fixed-end beam, and the vibration units are distributed within a square with a side length of about 4 mm. Figure 7b displays the packaged piezoelectric MEMS speaker chip. It can be seen that the chip is fixed on the PCB and packaged in a 3D-printed housing, and a silicone cover is selected as an earplug. The surface roughness of the PZT is shown in Figure 7c, with a root-mean-square roughness (R_q_) of 0.948 nm, revealing a relatively smooth PZT surface. The cross-sectional SEM image of the vibration membrane is shown in Figure 7d, clearly displaying the PZT and mica layers, with the thickness of the mica layer being about 25 μm. In Figure 7e, the functional layer PZT is clearly displayed, with a thickness of approximately 1 μm. Meanwhile, it can be seen that the top electrode Pt, the functional layer PZT, and the bottom electrode form a sandwich structure.

### 3.2. Measured Vibration Displacement

**Figure 8:**
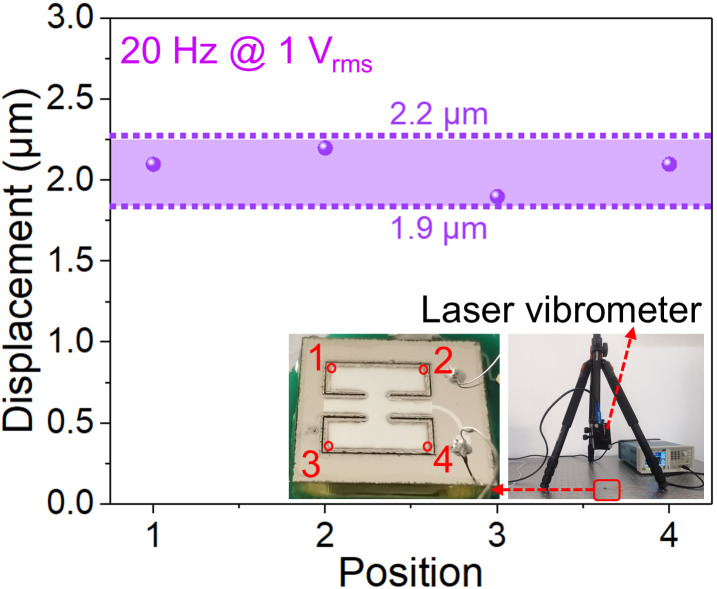
The laser vibrometer and vibration displacement.

As shown in Figure 8, a laser vibrometer was used to measure the vibration displacement at four points on the vibration membrane of the piezoelectric MEMS speaker. Under a driving voltage of 1 V_rms_, the measured vibration displacement ranges between 1.9 and 2.2 μm, which is somewhat different from that of the simulation of 2.4 μm. This difference is mainly attributed to the discrepancies between the actual structural parameters and the idealized ones used in the simulation.

### 3.3. Measured SPL

**Figure 9:**
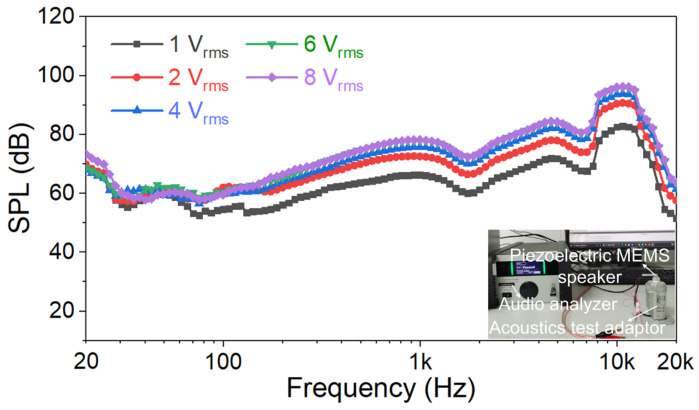
Measured SPL curves of the fixed-end beam–cantilever piezoelectric MEMS speaker at different driving voltages.

Given that the SPL of piezoelectric MEMS speakers remains relatively low and their current application scenario is limited to in-ear headphones, the acoustic tests were conducted in an acoustic test adapter rather than a large anechoic chamber. The SPL tests were conducted in a laboratory equipped with sound-insulating glass, especially at night, to ensure that the noise level was ≤25 dB. As shown in Figure 9, the reference measurement microphone used was a Class 1 measurement-grade M2010, which can be directly placed inside the cavity of the acoustic test adapter. Measurements were taken in an acoustic test adapter, with the sound outlet of the test piezoelectric MEMS speaker directly aligned and inserted into the adapter. The driving voltage signal was a swept-frequency signal. Under a driving voltage of 1 V_rms_, the SPL exceeds 51.6 dB within the frequency range of 20 Hz to 20 kHz, meeting the requirement for in-ear applications. The SPL curve shows that the resonance frequency is approximately 10.6 kHz, which has a certain discrepancy with the 11 kHz displayed in the simulation of the generic 711 coupler, mainly due to errors in the 3D-printing shell and encapsulation. As the driving voltage increases from 1 V_rms_ to 8 V_rms_, the SPL fluctuates slightly within the frequency range of 20–100 Hz and shows a trend of gradually increasing and then slowly stabilizing between 100 Hz and 20 kHz. And the SPL is increased from 82.8 dB, 90.7 dB, 93.8 dB, 95.9 dB to 96.2 dB at 10.6 kHz. A mica sheet exhibits the macroscopic mechanical behavior of flexible materials and the stiffness characteristics of rigid materials. Although a mica sheet possesses a certain degree of flexibility, its Young’s modulus is approximately 60 GPa, which is comparable to that of PZT; both are thus classified as rigid materials in terms of stiffness. The rigid structural design itself inherently leads to fluctuations in SPL. First, the frequency response curve of a rigid structure tends to exhibit significant peaks and valleys. In addition, the energy conversion efficiency of the inverse piezoelectric effect exhibits an inhomogeneous distribution with frequency. Meanwhile, microscale structures are highly sensitive to processing/material errors. These three factors ultimately result in significant fluctuations in SPL across the entire frequency band. In addition, the SPLs obtained from actual testing are lower than those from the simulation, mainly because of the idealization of simulation conditions and the simplification of complex structures.

### 3.4. THD and Steepness

**Figure 10:**
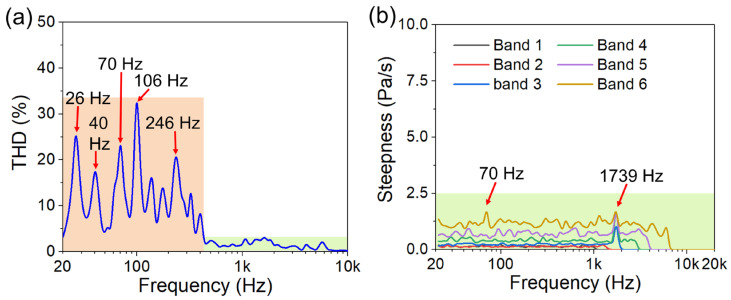
THD and steepness of the fixed-end beam–cantilever piezoelectric MEMS speaker under a driving voltage of 1 V_rms_: (**a**) THD of the piezoelectric MEMS speaker; (**b**) steepness of the piezoelectric MEMS speaker.

As shown in Figure 10a, when the driving voltage is 1 V_rms_, the total harmonic distortion (THD) is less than 3.4% at frequencies higher than 430 Hz, which meets the basic commercialization requirements. However, the THD is relatively high in the frequency range of 20 Hz to 430 Hz, especially at the low frequencies of 26 Hz, 40 Hz, 70 Hz, 106 Hz, and 246 Hz. This high THD is primarily attributed to the nonlinear strain of the vibration membranes, and the emergence of multiple THD peaks at low frequencies is attributed to the dipole rearrangement. PZT is a ferroelectric material containing a large number of domain walls. When the cantilever is driven, the combined action of the electric field and stress causes the domain walls to undergo reversible/irreversible displacement, multi-time-scale relaxation, and hopping between local energy potential wells. These processes are inherently nonlinear and can lead to distortion in the sound pressure output, resulting in peaks in the THD. More importantly, the domain-wall dynamics in PZT do not correspond to a single relaxation process but rather involve multiple modes, which give rise to multiple THD peaks. The technique of identifying abnormal sounds in speakers based on steepness is the PureSound detection technology developed by NTi Audio. The essence of abnormal sound is that the curve is no longer smooth, manifested as an abrupt change in sound. This mutation disrupts the harmony of the sound, leading to perceived abnormal sound. In the curve, this manifests as spikes or roughness [48]. As demonstrated in Figure 10b, the steepness in different frequency bands is significantly lower than 20 Pa/s within 20 Hz to 20 kHz under a driving voltage of 1 V_rms_, indicating that there is no abnormal sound caused by the collision between the vibration units, poor electrode connection, and loose fixation of the vibration membrane.

### 3.5. Fracture and Fatigue Resistance

**Figure 11:**
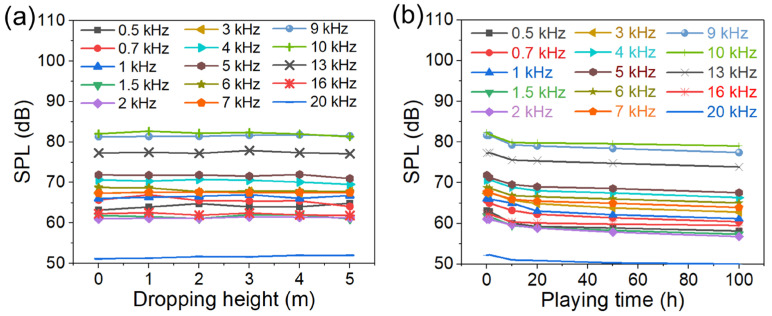
Fracture and fatigue resistance of the proposed fixed-end beam–cantilever piezoelectric MEMS speaker device: (**a**) fracture resistance; (**b**) fatigue resistance.

The existing studies lack reliability tests, especially drop tests [9,49]. The fracture resistance is characterized by comparing whether the SPL changes significantly after falling from different heights. As shown in Figure 11a, the SPL achieved after dropping from a height of 1 m, 2 m, 3 m, 4 m, or 5 m shows no significant difference compared to that before dropping, demonstrating its good fracture resistance. This is mainly due to the low modulus of elasticity and the flexibility of the flexible supporting layer. The fatigue resistance is evaluated by comparing whether the SPL changes significantly after continuous music playback for different durations. In Figure 11b, the device was subjected to continuous music playback for 1 h, 10 h, 20 h, 50 h, and 100 h, respectively. Compared with its initial state, the device maintained at least 92% of its original SPL even after 100 h of continuous music playback. Therefore, this also proves that the prepared piezoelectric MEMS speaker has good fatigue resistance, which is attributed to the low modulus of elasticity, layered structure, and certain flexibility of the mica sheet.

### 3.6. Comparison with Other Reported Works

As can be seen from Table 2, compared with the existing traditional three types of piezoelectric MEMS speakers [30,31,32], we have designed a brand-new fixed-end beam–cantilever type speaker, and adopted mica sheets as both the supporting layer and the insulation layer instead of the traditional Si supporting layer and SiO_2_ insulation layer. Compared with the traditional three types of piezoelectric MEMS speakers [30,31,32], the SPL of the proposed fixed-end beam–cantilever piezoelectric MEMS speaker with flexible supporting layer achieves a comparable SPL. In addition, experiments have confirmed that the proposed piezoelectric MEMS speaker exhibits excellent fracture resistance and fatigue resistance.

In summary, the main merits of this work are as follows. (a) This paper proposes a fixed-end beam–cantilever piezoelectric MEMS speaker, which can enhance the SPL through the superposition of structural vibration displacements, providing a new design perspective for piezoelectric MEMS speakers. (b) This proposed piezoelectric MEMS speaker adopts a flexible supporting layer that differs from the traditional Si supporting layer, theoretically enhancing SPL while also offering potential solutions for the future fabrication of piezoelectric MEMS speakers with fracture-resistance and fatigue-resistance properties. The main shortcomings of this work are as follows. (a) The SPL of the proposed fixed-end beam–cantilever piezoelectric MEMS speaker still fails to meet the requirements for commercialization. (b) The THD of the proposed fixed-end beam–cantilever piezoelectric MEMS speaker below 430 Hz does not meet the requirements for commercialization. Certainly, the performance of the proposed piezoelectric MEMS speaker utilizing the new fixed-end beam–cantilever structure and flexible supporting layer still requires further research and optimization.

In addition, this work does not discuss the relationship between electrode coverage and performance [50]. We will carry out a detailed study on the quantitative relationship between electrode coverage and performance in our follow-up research.

## 4. Conclusions

In summary, we present a fixed-end beam–cantilever piezoelectric MEMS speaker with a flexible supporting layer, which achieves a comparable SPL to that of the existing traditional three types of piezoelectric MEMS speakers based on a Si supporting layer and demonstrates good reliability. The packaged prototype device was measured after being installed in an acoustic test adapter. The results show that under a 1 V_rms_ driving voltage, the SPL exceeds 51.6 dB in the audible frequency range of 20 Hz–20 kHz, achieving an SPL comparable to that of traditional three types of piezoelectric MEMS speakers based on a Si supporting layer. The frequency range where THD meets the auditory requirements of the human ear is above 430 Hz, with all values below 3.4%. Furthermore, additional experiments confirmed its reliability by revealing that there is no abnormal sound output, no fracture after falling from a height of 1 to 5 m, and the device can still maintain more than 92% of its original SPL after playing music for 100 h. A fixed-end beam–cantilever structure is proposed, which can enhance the SPL through the superposition of structural vibration displacements. Instead of the conventional Si supporting layer, a mica sheet, which possesses a lower Young’s modulus but still maintains good flatness, is adopted. Compared with the Si supporting layer, the mica sheet can theoretically generate larger vibration displacement and higher SPL while maintaining good fracture resistance and fatigue resistance. Therefore, the proposed fixed-end beam–cantilever piezoelectric MEMS speaker based on a flexible supporting layer offers fresh insights for researchers and enterprises, potentially accelerating their development and practical implementation in the field.

## Figures and Tables

**Table 1:** Properties of different materials.

Materials	d_31_ (pm/V)	Young’s Modulus (GPa)	Density (kg/m^3^)	Poisson’s Ratio
PZT	−120	~65	7700	0.35
Mica	/	~60	2650	0.22
Pt	/	~170	21,400	0.38

**Table 2:** Comparison of our work with other reported works.

Reference	Structure	Supporting/Insulation Layer	SPL (20 Hz–20 kHz)	Reliability
[30]	Film type	Si/SiO_2_	≥60 (10 V_pp_)	/(No test)
[31]	Piston type	Si/SiO_2_	≥50 (2 V_pp_)	/(No test)
[32]	Cantilever type	Si/SiO_2_	≥59 (2 V_rms_)	/(No test)
**This work**	**Fix-end beam–cantilever type**	**Mica**	**≥51.6 (1 V_rms_); ≥59 (6 V_rms_)**	**√ (Test)**

## Data Availability

The data presented in this study are available on reasonable request from the author.
